# Environmental Impact of Conventional Versus Underwater Endoscopic Mucosal Resection for Colorectal Lesions: A Procedure‐Based Life Cycle Assessment With Pooled Effectiveness Estimates

**DOI:** 10.1002/deo2.70457

**Published:** 2026-10-26

**Authors:** Giulio Calabrese, Frederic Moll, João A. Cunha Neves, Livio Bonacci, Emanuele Sinagra, Fabiana Castiglione, Marcello Maida, Eduardo Albéniz‐Arbizu, Mathieu Pioche, Sandro Sferrazza

**Affiliations:** ^1^ Clinical Medicine and Surgery University of Naples Federico II Naples Italy; ^2^ Gastroenterology Unit San Pio Hospital Benevento Italy; ^3^ Gastroenterology and Endoscopy Unit Hôpital Edouard Herriot Lyon France; ^4^ Gastroenterology Department Unidade Local de Saúde do Algarve – Hospital de Portimão Portimão Portugal; ^5^ Gastroenterology and Endoscopy Unit ARNAS Civico Hospital Palermo Italy; ^6^ Endoscopy and Gastroenterology Unit Fondazione Istituto San Raffaele G. Giglio di Cefalù Cefalù Italy; ^7^ Department of Medicine and Surgery Kore University of Enna Enna Italy; ^8^ Gastroenterology Unit Ospedale Umberto I Enna Italy; ^9^ Department of Gastroenterology Hospital Universitario de Navarra Pamplona Spain; ^10^ Navarrabiomed Biomedical Research Center Universidad Pública de Navarra (UPNA) Pamplona Spain

**Keywords:** carbon footprint, colorectal lesions, green endoscopy, life cycle assessment, underwater EMR

## Abstract

**Objectives:**

The environmental impact of endoscopic techniques remains poorly defined. We compared procedure‐related environmental outcomes between conventional endoscopic mucosal resection (EMR) and underwater EMR (U‐EMR) for colorectal lesions.

**Methods:**

In this prospective pilot procedure‐based life cycle assessment of the resection phase, 10–25‐mm colorectal lesions underwent EMR or U‐EMR across three endoscopy units. Outcomes included global warming potential (GWP, kg CO_2_‐equivalent/procedure), waste, life‐cycle water consumption, fossil resource scarcity, and terrestrial acidification. Exploratory GWP per en bloc resection was estimated using pooled randomized‐trial and pilot‐cohort data.

**Results:**

Forty‐nine procedures were included: 23 EMR and 26 U‐EMR. *En bloc* resection was achieved in 56.5% of EMR and 69.2% of U‐EMR, and R0 resection in 52.2% and 69.2%. Mean GWP was significantly lower with U‐EMR than EMR (1.92 ± 0.54 vs. 2.42 ± 0.50 kg CO_2_‐eq; *p =* 0.002), corresponding to a reduction of 0.50 kg CO_2_‐eq per procedure and 50 kg CO_2_‐eq per 100 procedures. U‐EMR was associated with lower waste generation (324.0 ± 78.3 vs. 407.3 ± 72.0 g; *p* < 0.001), water consumption (0.56 ± 0.17 vs. 0.77 ± 0.20 m^3^; *p* < 0.001), fossil resource scarcity (0.33 ± 0.10 vs. 0.40 ± 0.09 kg oil‐eq; *p =* 0.007), and terrestrial acidification (0.0052 ± 0.0018 vs. 0.0068 ± 0.0018 kg SO_2_‐eq; *p =* 0.003). Findings were consistent across scenario and uncertainty analyses. In the meta‐analysis‐based model, GWP per en bloc resection was lower with U‐EMR than EMR (2.59 vs. 3.51 kg CO_2_‐eq), corresponding to a 26.3% relative reduction.

**Conclusions:**

U‐EMR was associated with lower procedure‐related environmental burden than conventional EMR, persisting after normalization for en bloc resection.

**Trial Registration:**

No. 165/26

## Graphical abstract

This prospective multicenter study compared the environmental impact of conventional and underwater endoscopic mucosal resection (EMR) for colorectal lesions using life cycle assessment. Underwater EMR was associated with significantly lower carbon emissions, waste generation, water consumption, and resource use. These environmental benefits persisted after accounting for procedural effectiveness using pooled data from randomized controlled trials. The findings suggest that underwater EMR may represent a more sustainable alternative to conventional EMR.

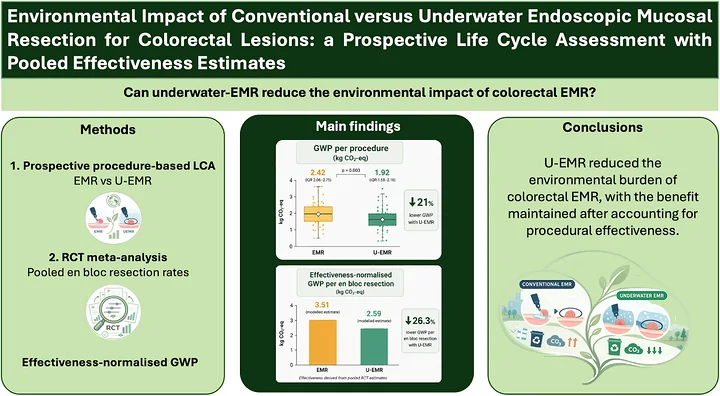

**Abbreviations**
CI: confidence interval
EMR: endoscopic mucosal resection
GWP: global warming potential
IQR: interquartile range
LCA: life cycle assessment
RCT: randomized controlled trial
RR: risk ratio
SD: standard deviation
U‐EMR: underwater endoscopic mucosal resection

## 1 Introduction

Gastrointestinal endoscopy contributes to healthcare‐related greenhouse gas emissions [1, 2], resource consumption, and clinical waste through energy and water use, reprocessing, single‐use devices, and procedural accessories [3, 4].

Green endoscopy therefore aims to reduce this burden while preserving quality, safety, and clinical effectiveness [5], with increasing awareness among endoscopists of the need to incorporate environmental considerations into clinical practice [6].

Although recent studies have quantified the carbon footprint of gastrointestinal endoscopy and identified its main environmental drivers [7, 8], comparisons between alternative techniques for the same indication remain limited.

Colorectal endoscopic mucosal resection (EMR) provides a relevant example. Unlike conventional EMR, which requires submucosal injection and ancillary materials, underwater EMR (U‐EMR) relies on water immersion to facilitate lesion capture and resection without injection [9]. Despite comparable outcomes in selected colorectal lesions [10], its environmental impact has not been assessed through life cycle assessment (LCA).

We therefore performed a prospective procedure‐based LCA comparing EMR and U‐EMR for colorectal lesions, complemented by pooled randomized trial data to provide effectiveness estimates for the exploratory global warming potential (GWP) per en bloc resection analysis.

## 2 Methods

### 2.1 Study Overview

This prospective pilot procedure‐based LCA compared the environmental impact of EMR and U‐EMR for colorectal lesions. To contextualize environmental impact according to procedural effectiveness, the study was complemented by a systematic review and pooled analysis of randomized controlled trials (RCTs) providing en bloc resection estimates for effectiveness‐normalized modelling.

### 2.2 Prospective Pilot Cohort

Consecutive patients undergoing EMR or U‐EMR for colorectal lesions were prospectively enrolled across three outpatient endoscopy units between November 2025 and March 2026. Eligible lesions measured 10–25 mm and were considered suitable for either technique. Lesions with features suggestive of deep submucosal invasion, recurrent or inflammatory bowel disease‐associated lesions, and procedures requiring planned combined or alternative resection techniques were excluded.

Technique selection was left to the treating endoscopist according to routine practice and technical feasibility, among lesions considered suitable for either technique or in the absence of predefined criteria favoring one technique over the other. Clinical, procedural, histological, and environmental data were prospectively collected using a standardized case‐report form.

Given the pilot design and absence of prior comparative procedure‐level LCA data, no formal sample size calculation was performed; all eligible procedures during the predefined period were included.

The study protocol was approved by the coordinating center ethics committee (CE Campania 3) (ID 165/26). The prospective observational component was reported in accordance with Strengthening the Reporting of Observational Studies in Epidemiology (STROBE) recommendations [11] (Table S1).

### 2.3 Procedures and Outcomes

All procedures were performed by experienced endoscopists using standard high‐definition colonoscopes. Conventional EMR was performed after submucosal injection, followed by snare resection, whereas U‐EMR was performed under water immersion without submucosal injection. Defect closure and hemostasis were performed at the treating endoscopist's discretion.

The main clinical effectiveness outcome was en bloc resection, defined as single‐specimen removal. Secondary clinical outcomes included R0 resection, procedure duration, intraprocedural bleeding, histology, and adverse events. R0 resection was defined as negative lateral and vertical histological margins.

Procedure duration was defined as the time from the beginning of lesion‐directed resection to completion of resection and defect management. Intraprocedural bleeding was recorded when endoscopic hemostasis was required during the procedure.

### 2.4 Environmental Inventory and System Boundaries

The environmental assessment was designed and reported according to the core principles of the Endoscopic Sustainability PrimAry Reporting Essentials framework [12]. The functional unit was one colorectal EMR procedure. The primary outcome was GWP per procedure (kg CO_2_‐equivalent); secondary outcomes were waste (g), life‐cycle water consumption (m^3^), fossil resource scarcity (kg oil equivalent), and terrestrial acidification (kg SO_2_‐equivalent).

The system boundary was restricted to the endoscopic procedure phase and included resources directly attributable to the resection technique, from preparation of resection materials in the endoscopy room to procedure completion.

Procedure‐related resources included disposable devices, consumables, fluids, dyes, sedation‐related medications, CO_2_ insufflation, water delivery systems, clips, and endoscopy tower time. The complete item‐level inventory is reported in Table S2. Resources not directly attributable to the resection technique, including patient and staff travel, bowel preparation, endoscope manufacturing and reprocessing, room heating, ventilation and air conditioning, histopathological processing, and post‐procedural care, were not included.

For each procedure, resource use was prospectively recorded using a standardized environmental inventory form. Activity data were primarily process‐based and procedure‐specific. Total waste generated during each procedure was measured directly after completion and expressed in grams per procedure.

### 2.5 Environmental Impact Calculation

Environmental impacts were calculated using openLCA version 2.5, with prospective procedure‐level foreground data and ecoinvent version 3.10 background data, supplemented when required by published LCA studies and product‐specific information. Impact assessment was performed using the ReCiPe 2016 midpoint (H) method for GWP, water consumption, fossil resource scarcity, and terrestrial acidification.

Detailed modelling assumptions, item‐level calculations, and conversion of GWP reductions into passenger‐car driving‐distance equivalents are provided in the Supporting Information Methods [13].

### 2.6 Systematic Review and Pooled Analysis for Effectiveness Estimates

A systematic review and pooled analysis of RCTs comparing EMR and U‐EMR for colorectal lesions was conducted according to Preferred Reporting Items for Systematic Reviews and Meta‐Analyses recommendations [14] (Table S3) and registered in PROSPERO (CRD420261374693). Search strategy, eligibility criteria, study selection, data extraction, risk‐of‐bias assessment, and meta‐analytic methods are provided in the Supporting Information Methods [15, 16, 17]. Pooled en bloc resection rates were used for effectiveness‐normalized modeling.

### 2.7 Effectiveness‐normalized Environmental Model

As an exploratory efficiency measure integrating environmental impact with procedural effectiveness, GWP per en bloc resection was calculated as mean GWP per procedure divided by the en bloc resection rate. The primary model used pooled rates from RCTs, while a sensitivity analysis used rates from the prospective pilot cohort. Absolute and relative differences between techniques were estimated, together with projected GWP savings per 100 en bloc resections.

### 2.8 Statistical, Scenario, and Uncertainty Analyses

Continuous variables were reported as mean ± standard deviation (SD) or median with interquartile range (IQR), and categorical variables as counts and percentages. Environmental outcomes were expressed as mean ± SD per procedure. Groups were compared using Student's *t*‐test or Mann–Whitney U test for continuous variables and chi‐square or Fisher's exact test for categorical variables, as appropriate.

Prespecified scenario, sensitivity, and probabilistic uncertainty analyses are detailed in the Supporting Information Methods.

Analyses were performed in R version 4.6.0 through RStudio. Two‐sided *p*‐values <0.05 were considered statistically significant.

## 3 Results

### 3.1 Study Population and Procedural Outcomes

Overall, 49 procedures in 49 patients were included, of which 23 were performed using conventional EMR and 26 using U‐EMR. Median lesion size was 20 mm (IQR 18–22), and median size, morphology, site, and access (SMSA) score was 9 (IQR 8–11). Lesions were located in the rectum in eight cases (16.3%), in the left colon in 12 cases (24.5%), and in the right colon in 29 cases (59.2%). Baseline demographic, lesion‐related, and procedural characteristics were broadly comparable between groups.

Procedure duration was comparable between EMR and U‐EMR [23 (IQR 15–30) vs. 18.5 (IQR 10–30) minutes, *p =* 0.308]. Median clip use was lower with U‐EMR than with EMR [2 (IQR 1–2) vs. 3 (IQR 1.5–3), *p =* 0.004]. *En bloc* resection was achieved in 31 procedures (63.3%), with rates of 56.5% after EMR and 69.2% after U‐EMR (*p =* 0.36). R0 resection was achieved in 30 procedures (61.2%), with rates of 52.2% after EMR and 69.2% after U‐EMR (*p =* 0.35). Intraprocedural bleeding requiring endoscopic hemostasis occurred in four cases (8.2%): 1/23 (4.3%) after EMR and 3/26 (11.5%) after U‐EMR (*p =* 0.36). Hemostasis was achieved using snare‐tip coagulation in all cases. No delayed bleeding or intra‐ or post‐procedural perforation occurred.

Table 1 summarizes all population characteristics and procedural outcomes.

**TABLE 1:** Baseline characteristics and procedural outcomes of the prospective pilot cohort.

Variable	Overall (*n =* 49)	EMR (*n =* 23)	U‐EMR (*n =* 26)	*p*
Median age, y [IQR]	65.00 [55.00, 75.00]	62.00 [51.00, 70.50]	68.00 [55.00, 75.00]	0.22
Median lesion size, mm [IQR]	20.00 [18.00, 22.00]	18.00 [15.00, 20.00]	20.00 [20.00, 22.00]	0.10
Median SMSA score [IQR]	9.00 [8.00, 11.00]	9.00 [8.00, 11.00]	9.50 [8.00, 11.00]	0.61
Female sex, *n* (%)	22 (44.9)	12 (52.2)	10 (38.5)	0.50
Location				
Rectum	8 (16.3)	4 (17.4)	4 (15.4)	0.85
Left colon	12 (24.5)	6 (26.1)	6 (23.1)	
Right colon	29 (59.2)	13 (56.5)	16 (61.5)	
Morphology				
Protruding	22 (44.9)	10 (43.5)	12 (46.2)	0.63
Granular	17 (34.7)	7 (30.4)	10 (38.5)	
Non‐granular	10 (20.4)	6 (26.1)	4 (15.4)	
Paris classification				
0‐Is	14 (28.6)	6 (26.1)	8 (30.8)	0.17
0‐IIa	20 (40.8)	11 (47.8)	9 (34.6)	
0‐IIb	6 (12.2)	2 (8.7)	4 (15.4)	
0‐IIa+Is	9 (18.4)	4 (17.4)	5 (19.2)	
Median procedure duration, min [IQR]	23.00 [10.00, 30.00]	23.00 [15.00, 30.00]	18.50 [10.00, 30.00]	0.308
Median number of clips [IQR]	2.00 [1.00, 3.00]	3.00 [1.50, 3.00]	2.00 [1.00, 2.00]	0.004
Sedation type, *n* (%)				
None	2 (4.1)	0 (0.0)	2 (7.7)	0.12
Conscious sedation	38 (77.6)	19 (82.6)	19 (73.1)	
Deep sedation	9 (18.4)	4 (17.4)	5 (19.2)	
*En bloc*, n (%)	31 (63.3)	13 (56.5)	18 (69.2)	0.36
R0, *n* (%)	30 (61.2)	12 (52.2)	18 (69.2)	0.35
Intraprocedural bleeding, *n* (%)	4 (8.2)	1 (4.3)	3 (11.5)	0.36
Histology, *n* (%)				
Hyperplastic polyps	3 (6.1)	0 (0.0)	3 (11.5)	0.043
Sessile serrated lesion	2 (4.1)	0 (0.0)	2 (7.7)	
Sessile serrated adenoma	9 (18.4)	5 (21.7)	4 (15.4)	
Villous adenoma	18 (36.7)	6 (26.1)	12 (46.2)	
Tubulovillous adenoma	17 (34.7)	12 (52.2)	5 (19.2)	
Dysplasia, *n* (%)				
None	3 (6.1)	0 (0.0)	3 (11.5)	0.227
LGD	37 (75.5)	19 (82.6)	18 (69.2)	
HGD	9 (18.4)	4 (17.4)	5 (19.2)	

Abbreviations: EMR, endoscopic mucosal resection; HGD, high‐grade dysplasia; IQR, interquartile range; LGD, low‐grade dysplasia; mm, millimeters; n, number; R0, complete resection with histologically negative margins; SMSA, size, morphology, site and access score; U‐EMR, underwater endoscopic mucosal resection; y, years.

### 3.2 Environmental Outcomes

Mean GWP per procedure was significantly lower for U‐EMR than for EMR [1.92 (SD 0.54) vs. 2.42 (SD 0.50) kg CO_2_‐eq, *p =* 0.002]. This corresponded to an absolute reduction of 0.50 kg CO_2_‐eq per procedure, equivalent to approximately 4.7 km driven by an average European passenger car. When projected over 100 procedures, the use of U‐EMR instead of conventional EMR would save approximately 50 kg CO_2_‐eq, corresponding to about 470 km driven. U‐EMR was also associated with significantly lower total waste generation [324.02 (SD 78.28) vs. 407.27 (SD 72.02) g/procedure, *p* < 0.001], water consumption [0.56 (SD 0.17) vs. 0.77 (SD 0.20) m^3^, *p* < 0.001], fossil resource scarcity [0.33 (SD 0.10) vs. 0.40 (SD 0.09) kg oil eq, *p =* 0.007], and terrestrial acidification [0.0052 (SD 0.0018) vs. 0.0068 (SD 0.0018) kg SO_2_‐eq, *p =* 0.003] (Table 2 and Figure 1).

**TABLE 2:** Procedure‐level environmental outcomes according to resection technique.

Variable	Overall (*n =* 49)	EMR (*n =* 23)	U‐EMR (*n =* 26)	*p*
Mean global warming potential per procedure, kg CO_2_‐eq (SD)	2.16 (0.57)	2.42 (0.50)	1.92 (0.54)	0.002
Mean total waste generated per procedure, g (SD)	363.10 (85.62)	407.27 (72.02)	324.02 (78.28)	<0.001
Mean water consumption, m^3^ (SD)	0.66 (0.21)	0.77 (0.20)	0.56 (0.17)	<0.001
Mean fossil resource scarcity, kg oil‐eq (SD)	0.36 (0.10)	0.40 (0.09)	0.33 (0.10)	0.007
Mean acidification, kg SO_2_‐eq (SD)	0.0059 (0.0020)	0.0068 (0.0018)	0.0052 (0.0018)	0.003

**FIGURE 1:**
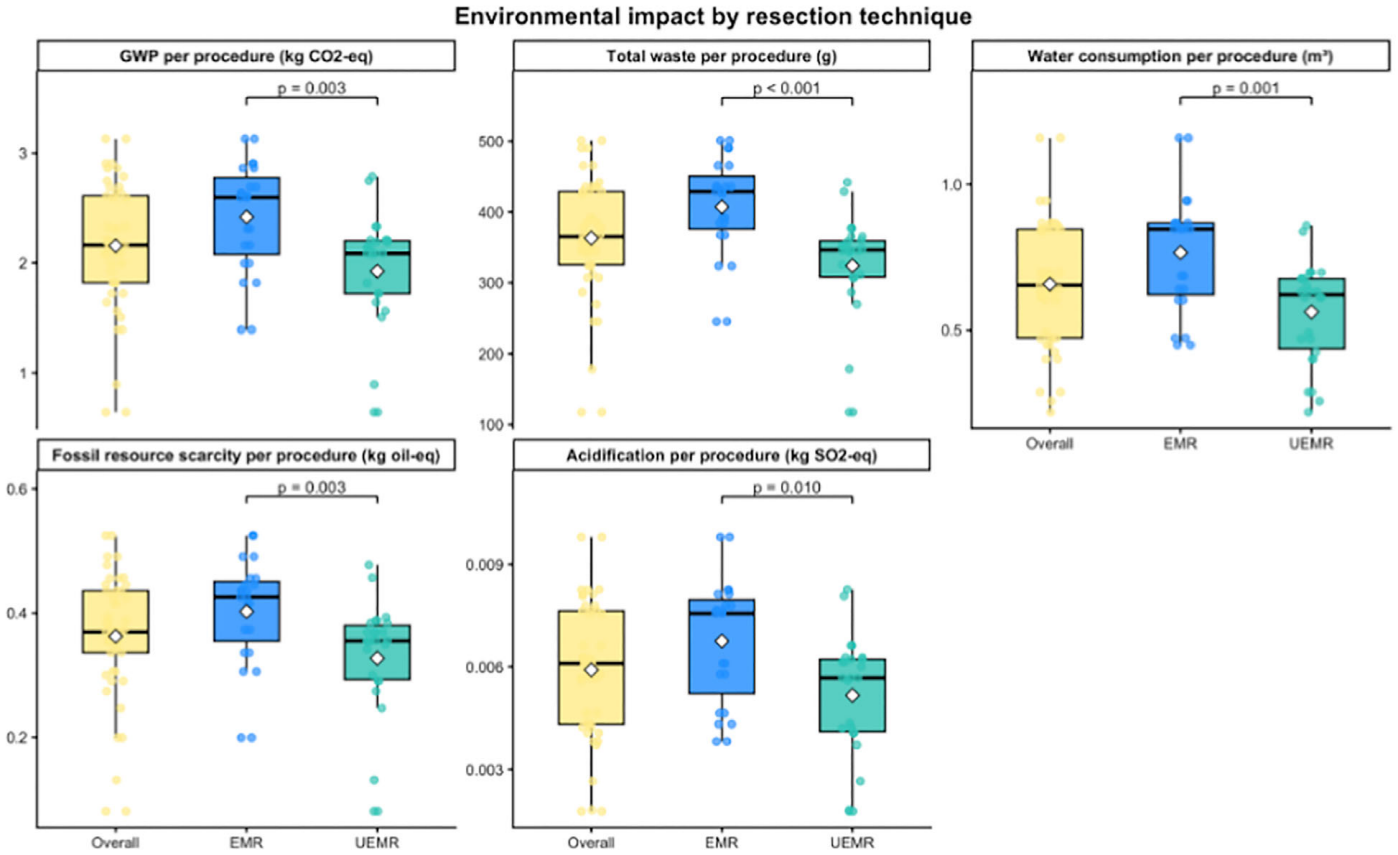
Procedure‐level environmental impact according to resection technique. Box plots show median and interquartile range; diamonds indicate mean values. *p*‐Values refer to Mann–Whitney U tests comparing EMR and U‐EMR. EMR, endoscopic mucosal resection; U‐EMR, underwater endoscopic mucosal resection; GWP, global warming potential.

Compared with U‐EMR, EMR involved injection needles and submucosal injection‐related materials, higher CO_2_ insufflation volume, longer endoscopy tower time, and more frequent clip use. U‐EMR, in contrast, required greater use of water delivery‐related items (Table S2).

In exploratory analyses stratified by lesion size and location, mean GWP was numerically lower with U‐EMR across all subgroups, although differences were not statistically significant within both size strata and the right‐colon subgroup (Table S4).

### 3.3 Scenario and Uncertainty Analyses

The lower environmental impact of U‐EMR remained consistent across the prespecified water‐use and CO_2_ scenario analyses (Table 3). Under the all‐water‐bag scenario, mean GWP remained lower with U‐EMR than with EMR [1.84 (SD 0.49) vs. 2.38 (SD 0.50) kg CO_2_‐eq, *p* < 0.001], as did total waste generation [316.02 (SD 74.08) vs. 404.47 (SD 71.35) g/procedure, *p* < 0.001]. Similar differences were observed under the all‐flask and low‐flow CO_2_ scenarios.

**TABLE 3:** Scenario analyses of procedure‐level environmental outcomes according to resection technique.

Scenario	GWPP Overall	GWPP EMR	GWPP U‐EMR	*p*
Observed	2.16 (0.57)	2.42 (0.50)	1.92 (0.54)	0.002
All water bags	2.10 (0.56)	2.38 (0.50)	1.84 (0.49)	<0.001
All flasks	2.17 (0.57)	2.42 (0.50)	1.95 (0.53)	0.003
Low‐flow CO2	2.15 (0.57)	2.41 (0.50)	1.92 (0.54)	0.002
No clips	1.30 (0.21)	1.37 (0.17)	1.23 (0.22)	0.021
Standardized 2‐clip	2.19 (0.21)	2.26 (0.17)	2.13 (0.22)	0.021
	**TWGP Overall**	**TWGP EMR**	**TWGP U‐EMR**	***p***
Observed	363.10 (85.62)	407.27 (72.02)	324.02 (78.28)	<0.001
All water bags	357.54 (84.74)	404.47 (71.35)	316.02 (74.08)	<0.001
All flasks	363.53 (84.97)	406.67 (72.43)	325.37 (77.59)	<0.001
Low‐flow CO_2_	363.13 (85.65)	407.33 (72.04)	324.02 (78.28)	<0.001
No clips	250.55 (42.77)	270.10 (35.25)	233.25 (41.91)	0.002
Standardized 2‐clip	368.55 (42.77)	388.10 (35.25)	351.25 (41.91)	0.002
	**TWC Overall**	**TWC EMR**	**TWC U‐EMR**	***p***
Observed	0.66 (0.21)	0.77 (0.20)	0.56 (0.17)	<0.001
All water bags	0.66 (0.21)	0.77 (0.20)	0.56 (0.17)	<0.001
All flasks	0.66 (0.21)	0.77 (0.20)	0.56 (0.17)	<0.001
Low‐flow CO_2_	0.66 (0.21)	0.77 (0.20)	0.56 (0.17)	<0.001
No clips	0.26 (0.05)	0.27 (0.05)	0.24 (0.03)	0.033
Standardized 2‐clip	0.67 (0.05)	0.69 (0.05)	0.66 (0.03)	0.036
	**TFRS Overall**	**TFRS EMR**	**TFRS U‐EMR**	***p***
Observed	0.36 (0.10)	0.40 (0.09)	0.33 (0.10)	0.007
All water bags	0.36 (0.10)	0.40 (0.09)	0.32 (0.09)	0.002
All flasks	0.36 (0.10)	0.40 (0.09)	0.33 (0.10)	0.007
Low‐flow CO_2_	0.36 (0.10)	0.40 (0.09)	0.33 (0.10)	0.007
No clips	0.24 (0.06)	0.25 (0.05)	0.22 (0.06)	0.122
Standardized 2‐clip	0.37 (0.06)	0.38 (0.05)	0.36 (0.06)	0.253
	**TAC Overall**	**TAC EMR**	**TAC U‐EMR**	***p***
Observed	0.00591 (0.00195)	0.00676 (0.00182)	0.00517 (0.00178)	0.003
All water bags	0.00569 (0.00191)	0.00662 (0.00181)	0.00487 (0.00162)	<0.001
All flasks	0.00601 (0.00192)	0.00678 (0.00182)	0.00532 (0.00177)	0.007
Low‐flow CO_2_	0.00591 (0.00195)	0.00676 (0.00183)	0.00517 (0.00178)	0.003
No clips	0.00244 (0.00033)	0.00248 (0.00029)	0.00240 (0.00036)	0.393
Standardized 2‐clip	0.00602 (0.00032)	0.00605 (0.00027)	0.00600 (0.00036)	0.564

Abbreviations: GWPP, global warming potential per procedure; TAC, Total acidification; TFRS, Total fossil resource scarcity; TWC, Total water consumption; TWGP, total waste generated per procedure.

After excluding clip‐related impacts, GWP remained lower with U‐EMR than with EMR [1.23 (SD 0.22) vs. 1.37 (SD 0.17) kg CO_2_‐eq/procedure, *p =* 0.021], while differences in fossil resource scarcity and terrestrial acidification were no longer significant. Similar findings were observed when clip use was standardized to two clips per procedure in both groups.

Monte Carlo simulation confirmed the robustness of the primary environmental estimates (Table S5). The mean estimated difference in GWP between EMR and U‐EMR was 0.49 kg CO_2_‐eq per procedure, with a 95% uncertainty interval of 0.41 to 0.58. The uncertainty intervals also consistently favored U‐EMR for total waste generation, water consumption, fossil resource scarcity, and terrestrial acidification.

### 3.4 Pooled‐analysis and Effectiveness‐normalized GWP

The systematic search identified 114 records. After screening and eligibility assessment, seven RCTs [18, 19, 20, 21, 22, 23, 24] comparing EMR and U‐EMR were included in the quantitative synthesis. Overall, these trials included 1633 colorectal lesions, of which 797 were treated by EMR and 836 by U‐EMR. Study flow is reported in Figure S1.

Risk‐of‐bias assessment using RoB 2 showed low risk of bias or some concerns across the included trials, with no study judged to be at high risk of bias (Figure S2A,B).

The certainty of evidence for en bloc resection was rated as moderate, mainly because of moderate between‐study heterogeneity, with no major concerns regarding risk of bias or indirectness.

In random‐effects meta‐analysis, U‐EMR was not associated with a statistically significant increase in en bloc resection compared with EMR (risk ratio [RR] 1.05, 95% confidence interval [CI] 0.98–1.13; *I* ^2^ = 53.1%, heterogeneity *p =* 0.0465) (Figure S3A). U‐EMR was associated with a higher probability of R0 resection compared with EMR (RR 1.22, 95% CI 1.01–1.49; *I* ^2^ = 60.6%, heterogeneity *p =* 0.0546) (Figure S3B). Conversely, no significant difference was observed for intraprocedural bleeding (RR 0.81, 95% CI 0.41–1.58; *I* ^2^ = 47.8%, heterogeneity *p =* 0.0880) (Figure S3C).

Study characteristics are summarized in Table S6.

In the exploratory effectiveness‐normalized analysis, when GWP was normalized to en bloc resection using pooled effectiveness estimates from RCTs, U‐EMR remained associated with a lower environmental burden than EMR (Figure 2; Table S7). In this primary meta‐analysis‐based model, GWP per en bloc resection was 2.59 kg CO_2_‐eq for U‐EMR and 3.51 kg CO_2_‐eq for EMR, corresponding to an absolute reduction of 0.92 kg CO_2_‐eq per en bloc resection and a relative reduction of 26.3%. When projected over 100 en bloc resections, this corresponded to an estimated saving of 92.39 kg CO_2_‐eq.

**FIGURE 2:**
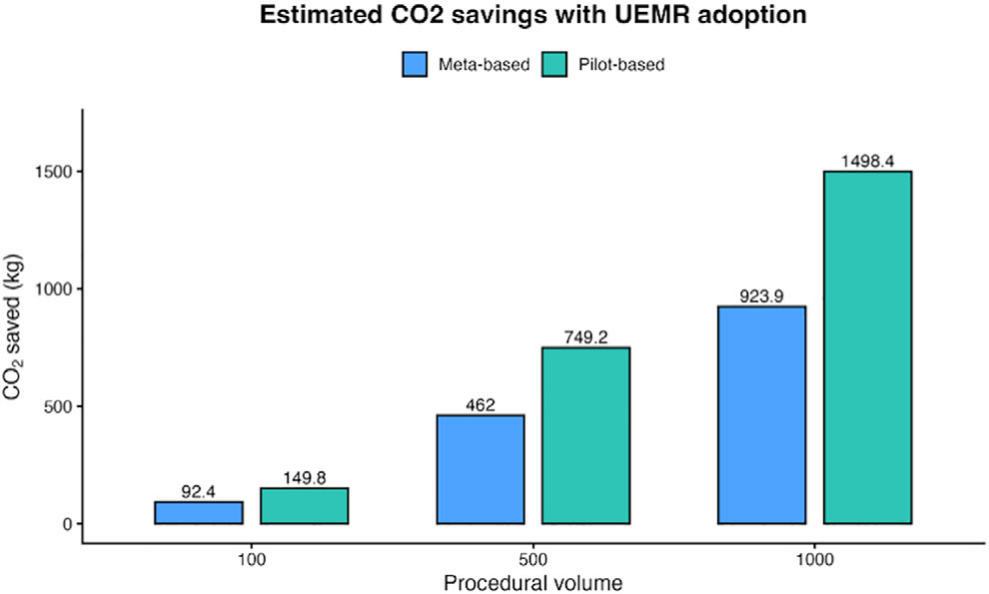
Effectiveness‐normalized global warming potential according to resection technique. Global warming potential (GWP) was expressed per en bloc resection using pooled en bloc resection rates from randomized controlled trials as the primary effectiveness input. Underwater endoscopic mucosal resection (U‐EMR) maintained a lower GWP per en bloc resection than conventional EMR, indicating that the environmental benefit persisted after accounting for broadly comparable clinical effectiveness.

In the pilot‐based sensitivity analysis, GWP per en bloc resection was 2.78 kg CO_2_‐eq for U‐EMR and 4.28 kg CO_2_‐eq for EMR, corresponding to an absolute reduction of 1.50 kg CO_2_‐eq per en bloc resection and a relative reduction of 35.0%. When projected over 100 en bloc resections, this corresponded to an estimated saving of 149.84 kg CO_2_‐eq.

## 4 Discussion

In this procedure‐based LCA, U‐EMR was associated with a lower environmental burden than conventional EMR for colorectal lesions across all prespecified outcomes, including GWP, total waste generation, water consumption, fossil resource scarcity, and terrestrial acidification. Findings remained consistent across scenario and probabilistic uncertainty analyses and were mainly driven by lower material requirements with U‐EMR. Although the absolute per‐procedure reduction was modest, its projection over 100 procedures illustrates how small differences may become meaningful in high‐volume practice. The environmental advantage of U‐EMR also persisted after normalization for en bloc resection using both pooled randomized‐trial estimates and pilot‐cohort rates.

These findings are plausibly explained by differences in material requirements and procedural workflow. Avoiding submucosal injection reduces single‐use consumables and may also decrease CO_2_ insufflation and endoscopy tower time. Conversely, U‐EMR requires more sterile water or saline containers. However, life‐cycle water consumption reflects water use across the life cycle of all included materials, rather than only procedural water use. These requirements were outweighed by consumables used more frequently or exclusively in conventional EMR, mainly indigo carmine, clips, and injection needles. U‐EMR therefore maintained a lower environmental impact even when assuming exclusive use of water flasks rather than water bags.

Clip use may have contributed substantially to the observed environmental difference. U‐EMR was associated with fewer clips than conventional EMR, in line with previous randomized data [18, 23], with clip‐related GWP accounting for approximately 1.07 and 0.69 kg CO_2_‐eq per procedure, respectively. After excluding clip‐related impacts or standardizing clip use, the GWP difference was attenuated but remained significant, suggesting that clip use contributed to, but did not fully explain, the observed difference. Underwater closure could potentially reduce clip requirements by decreasing mucosal tension and facilitating defect approximation [25]. However, clip use may also reflect lesion characteristics, bleeding risk, operator preference, and local closure policies and cannot be fully attributed to U‐EMR.

Randomized trials have supported U‐EMR as a valid alternative to conventional EMR for selected colorectal lesions, with broadly comparable effectiveness and safety outcomes [18, 21, 23]. In our pooled analysis, en bloc resection did not differ significantly between techniques, while U‐EMR was associated with a modestly higher probability of R0 resection; no significant difference was observed for intraprocedural bleeding, although our pilot cohort was underpowered for safety outcomes. Environmental impact may therefore inform selection among clinically comparable techniques.

Although our pilot cohort was not powered to assess clinical non‐inferiority, the exploratory effectiveness‐normalized analysis provides a hypothetical framework for relating environmental burden to procedural effectiveness.

Our findings fit within the emerging concept of comparative environmental effectiveness in therapeutic endoscopy. Sustainability studies have quantified emissions, waste, resource use, reprocessing pathways, device use, and unit‐level workflows. Recently, a post hoc carbon footprint analysis of the RESECT‐COLON randomized trial showed that environmental outcomes can be incorporated into comparative evaluations of therapeutic strategies [26]. By applying a prospective procedure‐level LCA to two commonly used resection techniques, our study extends this concept to selected colorectal lesions and supports a shift from describing the footprint of endoscopy to comparing alternative techniques for the same indication. This approach is consistent with calls for standardized endoscopy‐specific environmental metrics to enable benchmarking across units and studies [27]. Sustainability metrics could ultimately complement efficacy, safety, feasibility, cost, and resource availability when guidelines address clinically comparable techniques.

Some limitations should be acknowledged. Treatment allocation was not randomized, and the cohort was small; clinical comparisons and pilot‐derived effectiveness estimates should therefore be considered exploratory. Environmental estimates depended on predefined system boundaries, local workflows, material proxies, and modelling assumptions. Resources not directly attributable to the resection technique, including travel, bowel preparation, endoscope manufacturing and reprocessing, ventilation, histopathology, and post‐procedural care, were excluded. Downstream consequences such as recurrence, surveillance colonoscopies, and retreatment were not modelled because randomized evidence does not demonstrate consistent differences in longer‐term outcomes between EMR and U‐EMR. Finally, pooled randomized‐trial estimates may not fully reflect differences in lesion characteristics, operator practice, and closure policies across settings, limiting the clinical applicability of the effectiveness‐normalized estimates to our 10–25‐mm cohort.

In conclusion, this prospective pilot LCA showed that U‐EMR was associated with a lower resection‐phase environmental burden than conventional EMR for selected colorectal lesions, partly related to differences in injectate‐related materials and clip use, while persisting when clip‐related impacts were excluded or standardized. When both endoscopic techniques are clinically comparable, environmental outcomes may complement traditional decision‐making criteria. This appears particularly relevant given growing awareness of green endoscopy among endoscopists, while environmental considerations still appear to play a limited role in device selection [6]. Larger studies incorporating safety outcomes, recurrence, and downstream healthcare utilization are needed to confirm these findings across different settings, lesion types, and procedural workflows.

## Artificial Intelligence Use Disclosure

During the preparation of this manuscript, the authors used ChatGPT (OpenAI) for language editing and improving clarity and readability. All artificial intelligence‐assisted text was reviewed and verified by the authors, who take full responsibility for the final content.

## Author Contributions

**Giulio Calabrese**: conceptualization, data curation, formal analysis, methodology, Writing – original draft, and Writing – review & editing. **Frederic Moll**: data curation and writing – review & editing. **João A. Cunha Neves**: writing – review & editing. **Livio Bonacci**: data curation. **Emanuele Sinagra**: writing – review & editing. **Fabiana Castiglione**: writing – review & editing. **Marcello Maida**: writing – review & editing. **Eduardo Albéniz‐Arbizu**: validation and writing – review & editing. **Mathieu Pioche**: methodology, validation, writing – review & editing, and supervision. **Sandro Sferrazza**: conceptualization, writing – original draft, writing – review & editing, and guarantor of the article.

## Funding

The authors have nothing to report.

## Ethics Statement

The prospective study was conducted in accordance with the Declaration of Helsinki and was approved by the Ethics Committee Campania 3 (approval No. 165/26).

## Consent

Written informed consent was obtained from all participants enrolled in the prospective study.

## Conflicts of Interest

João A. Cunha Neves: consultancy to Boston Scientific; Fabiana Castiglione: advisory board for AbbVie, MSD, Takeda, Janssen, and Pfizer; Eduardo Albéniz‐Arbizu: consultancy to Creo Medical and AGS MedTech; honoraria for lectures, teaching activities, or as travel grants from Olympus, Boston, Fujifilm, Norgine, Casen Recordati, and 3D Matrix; Mathieu Pioche: teaching activities for Olympus, Cook, and Boston Scientific; Sandro Sferrazza: consultancy to Fujifilm; All other authors declare no conflicts of interest.

## Supporting information




**Supporting File 01**: deo270457‐sup‐0001‐FigureS1.tiff

**Supporting File 02**: deo270457‐sup‐0002‐FigureS2.tiff

**Supporting File 03**: deo270457‐sup‐0003‐FigureS3_R1.tiff

**Supporting File 04**: deo270457‐sup‐0004‐Table.docx

Supporting File 05: deo270457‐sup‐0005‐SupMat‐Methods.docx

## Data Availability

The data supporting the findings of this study are available from the corresponding author upon reasonable request.
