# Supplementary material for: Environmental Impact of Conventional Versus Underwater Endoscopic Mucosal Resection for Colorectal Lesions: A Procedure‐Based Life Cycle Assessment With Pooled Effectiveness Estimates
Source: DEN Open. 2026 Oct 26;7(1):e70457. doi: 10.1002/deo2.70457 (PMC13710893; doi:10.1002/deo2.70457)
Supplement: Supplementary file 1 [file DEO2-7-e70457-s004.docx]

**SUPPLEMENTARY METHODS**

**Environmental impact calculation**

Each inventory item was modelled according to its procedure-level quantity, unit weight, material composition, and relevant emission or characterisation factor. Weight was measured or obtained from product specifications, while composition was assigned from product information, packaging details, or the closest available proxy. Item-level contributions were summed for each procedure, applying identical databases, factors, allocation rules, and assumptions to both groups. Tower impact was estimated from procedure duration, assumed energy consumption, and the corresponding electricity factor; recorded CO₂ use was converted into kg CO₂-equivalent, and waste was measured directly.

GWP reductions were converted into passenger-car driving-distance equivalents using the European Environment Agency emission factor for new cars registered in the European Union in 2023, 106.4 g CO₂/km

**Systematic review and pooled analysis**

A systematic review with pooled analysis was performed to obtain external estimates of *en bloc* resection rates after conventional EMR and U-EMR for colorectal lesions. The review was conducted and reported according to the Preferred Reporting Items for Systematic Reviews and Meta-Analyses (PRISMA) recommendations.

**Search strategy**

A systematic literature search was performed in PubMed/MEDLINE, Embase, and the Cochrane Central Register of Controlled Trials from database inception to March 2026. The search strategy combined terms related to colorectal lesions, endoscopic mucosal resection, underwater endoscopic mucosal resection, and randomised controlled trials. No restriction on publication year was applied. Reference lists of included studies and relevant reviews were also screened manually to identify additional eligible studies.

The PubMed search strategy was as follows:

(colorectal OR colon OR rectal OR colorectal lesion* OR colorectal polyp* OR adenoma*) AND (“endoscopic mucosal resection” OR EMR) AND (underwater OR “underwater endoscopic mucosal resection” OR UEMR OR U-EMR) AND (randomized OR randomised OR random* OR trial)

The search strategy was adapted for each database.

**Eligibility criteria**

Studies were eligible if they met all of the following criteria: (1) randomised controlled trial design; (2) inclusion of patients undergoing endoscopic resection of colorectal lesions; (3) comparison between conventional EMR and U-EMR; (4) reporting of *en bloc* resection rates, or sufficient data to calculate them, separately for each treatment arm; and (5) publication in English.

Studies were excluded if they were non-randomised, observational, retrospective, case series, conference abstracts without sufficient outcome data, reviews, editorials, letters without original data, animal studies, non-English-language articles, or studies not directly comparing EMR and U-EMR. Studies evaluating other resection techniques without a clearly extractable EMR-versus-U-EMR comparison were also excluded.

**Study selection and data extraction**

After removal of duplicates, titles and abstracts were screened for eligibility. Full texts of potentially relevant articles were then assessed against the predefined inclusion and exclusion criteria. Study selection was performed independently by two reviewers (GC and MM), with disagreements resolved by discussion or by consultation with a third reviewer (SS).

For each included study, the following data were extracted using a standardised data extraction form: first author, year of publication, country, study design, number of patients and lesions, lesion size, lesion location, inclusion and exclusion criteria, intervention arm, comparator arm, number of lesions treated in each arm, number of en bloc resections, and other reported clinical outcomes when available.

**Outcome definition**

The primary outcome was en bloc resection, defined as removal of the target lesion in a single specimen. Secondary outcomes were R0 resection and intraprocedural bleeding, as defined in the individual trials. When both patient-level and lesion-level outcomes were reported, lesion-level data were preferentially extracted, consistent with the procedure-based functional unit of the environmental analysis.

**Risk-of-bias assessment**

Risk of bias in included randomised controlled trials was assessed using the revised Cochrane risk-of-bias tool for randomised trials (RoB 2). The following domains were evaluated: bias arising from the randomisation process; bias due to deviations from intended interventions; bias due to missing outcome data; bias in the measurement of the outcome; and bias in the selection of the reported result. Each study was classified as having low risk of bias, some concerns, or high risk of bias.

The certainty of evidence for en bloc resection was assessed using the GRADE approach, with randomised controlled trials initially considered as high-certainty evidence and rated down, when appropriate, for risk of bias, inconsistency, indirectness, imprecision, and publication bias.

**Data synthesis and meta-analysis**

For comparative clinical outcomes, risk ratios and 95% confidence intervals were pooled for en bloc resection, R0 resection, and intraprocedural bleeding using random-effects meta-analysis. Between-study heterogeneity was assessed using the I² statistic.

Because the effectiveness-normalised environmental model required absolute effectiveness estimates, en bloc resection rates were also pooled separately for the EMR and U-EMR arms. These arm-specific pooled rates were used exclusively as effectiveness inputs in the meta-analysis-based environmental model and were not used to estimate the comparative effect between the two techniques.

**Statistical, scenario, and uncertainty analyses**

Prespecified scenario analyses standardised water-bag use, water-flask use, and low-flow CO₂ insufflation, while all other assumptions remained unchanged. Additional sensitivity analyses assessed the influence of differential clip use by excluding clip-related environmental impacts and by standardising clip use to two clips per procedure in both groups. Probabilistic uncertainty in key emission and characterisation factors was assessed using 10,000-iteration Monte Carlo simulation and summarised by mean, median, standard deviation, coefficient of variation, standard error, and 95% uncertainty intervals.
